# Impact of Mucositis on Absorption and Systemic Drug Exposure of Isavuconazole

**DOI:** 10.1128/AAC.00101-17

**Published:** 2017-05-24

**Authors:** Laura L. Kovanda, Francisco M. Marty, Johan Maertens, Amit V. Desai, Christopher Lademacher, Marc Engelhardt, Qiaoyang Lu, William W. Hope

**Affiliations:** a Antimicrobial Pharmacodynamics and Therapeutics, Department of Molecular and Clinical Pharmacology, Institute of Translational Medicine, University of Liverpool, Liverpool, United Kingdom; b Astellas Pharma Global Development, Inc., Northbrook, Illinois, USA; c Brigham and Women's Hospital, Boston, Massachusetts, USA; d Universitaire Ziekenhuizen, Leuven, Belgium; e Basilea Pharmaceutica International Ltd., Basel, Switzerland

**Keywords:** antifungal agents, aspergillosis, invasive fungal disease, mucormycosis, mucositis, population pharmacokinetics, triazole

## Abstract

Isavuconazonium sulfate is the water-soluble prodrug of isavuconazole. Population analyses have demonstrated relatively predictable pharmacokinetic (PK) behavior in diverse patient populations. We evaluated the impact of mucositis on the oral isavuconazole exposure using population PK modeling. This study included patients treated in two phase 3 trials of isavuconazole, SECURE for treatment of invasive aspergillosis (IA) and other filamentous fungi and VITAL for patients with mucormycosis, invasive fungal disease (IFD) caused by other rare fungi, or IA and renal impairment. Mucositis was reported by site investigators and its impact on oral bioavailability was assessed. Use of the oral formulation was at the discretion of the investigator. Patients with plasma samples collected during the use of isavuconazonium sulfate were included in the construction of population PK model. Of 250 patients included, 56 patients had mucositis at therapy onset or as an adverse event during oral isavuconazole therapy. Levels of oral bioavailability were comparable, at 98.3% and 99.8%, respectively. The average drug exposures (average area under the curve [AUC_ave_]) calculated from either the mean or median parameter estimates were not different between patients with and without mucositis. Mortality and overall clinical responses were similar between patients receiving oral therapy with and without mucositis. We found that isavuconazole exposures and clinical outcomes in this subset of patients with mucositis who were able to take oral isavuconazonium sulfate were comparable to those in patients without mucositis, despite the difference in oral bioavailability. Therefore, mucositis may not preclude use of the oral formulation of isavuconazonium sulfate.

## INTRODUCTION

Invasive mold diseases (IMDs) are life-threatening conditions that require timely and intensive treatment. Patients with hematological disorders or who have undergone hematopoietic stem cell transplantation (HSCT) are a leading risk group for IMDs. Antineoplastic chemotherapy for acute myeloid leukemia (AML) or acute lymphocytic leukemia (ALL) and conditioning regimens for HSCT often cause mucosal disruption of the gastrointestinal (GI) tract (i.e., mucositis) that may compromise oral bioavailability (1). An evaluation of the impact of mucositis on the oral absorption of antifungal agents is required to ensure optimal antifungal therapy (2).

Isavuconazonium sulfate, the water-soluble prodrug of the triazole antifungal agent isavuconazole, is approved by the U.S. FDA in adults for the treatment of invasive aspergillosis (IA) and invasive mucormycosis (IM) and by the European Medicines Agency (EMA) for the treatment of IA and for IM in patients for whom amphotericin B is inappropriate (3, 4). The clinical formulations include both intravenous (i.v.) and oral capsules. The pharmacokinetics (PK) have been well characterized from substudies embedded in clinical trials (5–8). The pivotal clinical trials included more than 400 patients with >60% with hematological malignancies or other conditions that required intensive chemotherapy and the potential for mucositis (9, 10).

In this study, we examined the impact of mucositis on the bioavailability and drug exposure following the administration of oral isavuconazonium sulfate. We fitted a population pharmacokinetic model to the plasma concentrations from patients receiving oral isavuconazole in patients with and without mucositis and used this model to estimate oral bioavailability and the ultimate drug exposure. We consider the potential impact for dosing and therapeutic drug monitoring of isavuconazole in the setting of mucositis.

## RESULTS

### Study population.

**FIG 1:**
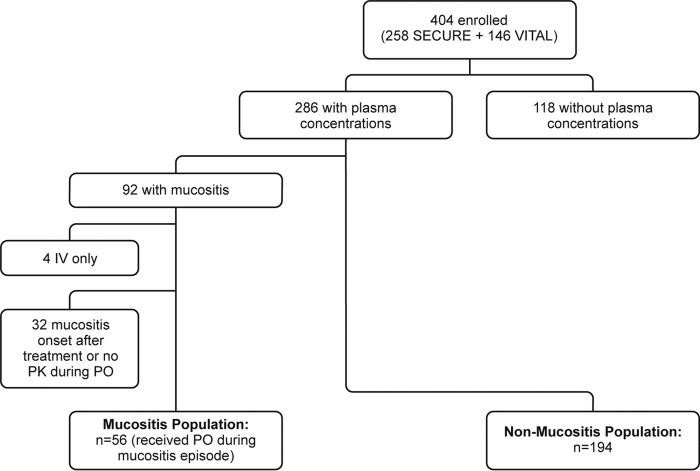
Flowchart illustrating flow of isavuconazole-treated patients into the mucositis and nonmucositis populations. PO, orally.

A total of 250 patients were included in the analysis, of whom 56 had mucositis. Figure 1 shows the flow of patient inclusion in the study. The majority of the mucositis patients had a hematologic malignancy (89.3%) that was active at the time of enrollment and were neutropenic at the start of antifungal treatment (78.2%) (Table 1). Only 6 patients did not have a hematological malignancy (aplastic anemia [*n* = 3], uterine leiomyosarcoma [*n* = 1], X-linked adrenomyeloneuropathy [*n* = 1], and squamous cell carcinoma of the tongue [*n* = 1]). A quarter (26.8%) of the patients with mucositis had received an HSCT. Sixteen percent of mucositis patients had baseline renal impairment (estimated glomerular filtration rate-modification of diet in renal disease [eGFR-MDRD] <60 ml/min/1.73 m^2^), compared with 27.6% of those without mucositis. The majority of the overall population were males (62%) and Caucasian (78.8%), and the average age (±standard deviation [SD]) and weight (±SD) were 50.3 ± 16.1 years and 70.0 ± 18.3 kg, respectively.

**TABLE 1:** Demographics, background disease, and duration of therapy

Characteristic	Value for:		
	Mucositis patients (*n* = 56)	Nonmucositis patients (*n* = 194)	Total (*n* = 250)
Age (yrs), median (minimum–maximum)	50 (18–79)	52 (19–92)	52 (18–92)
Male sex, no. (%)	32 (57)	123 (63)	155 (62)
Race, no. (%)			
White	48 (86)	149 (77)	197 (79)
Asian	7 (13)	31 (16)	38 (15)
Black	1 (2)	9 (5)	10 (4)
Other	0	5 (3)	5 (2)
			
Wt (kg), mean ± SD	71.7 ± 18.1	69.5 ± 18.4	70.0 ± 18.3
Underlying disease or condition, no. (%)			
Hematological malignancy	50 (89.3)	101 (52.1)	151 (60.4)
Active malignancy	40 (71.4)	76 (39.2)	116 (46.4)
Allogeneic HSCT	15 (26.8)	33 (17.0)	48 (19.2)
Baseline neutropenia	43 (78.2)	64 (41.8)	107 (51.4)
T-cell immunosuppressants	23 (41.8)	82 (51.9)	105 (49.3)
Use of corticosteroids	8 (14.3)	47 (24.2)	55 (22.0)
Duration of therapy (days), median (range)			
Total duration	75.5 (8–735)	83 (1–882)	82 (1–882)
i.v. formulation	9 (2–45)	7 (0.5–77)	7.5 (0.5–77)
Oral formulation	58 (1–690)	79.8 (0.5–882)	73 (0.5–882)

### Types of fungal infection in patients with mucositis.

Thirty-two patients had proven or probable IA, and 7 patients had possible IA (with appropriate host factors and clinical features but no mycological evidence of disease). Eight patients had proven or probable infection caused by various mold and rare yeasts, including Mucorales (*n* = 1), Fusarium spp. (*n* = 3), Culvularia lunata (*n* = 1), Alternaria spp. (*n* = 1), Acremonium spp. (*n* = 1), and Trichosporon spp. (*n* = 1). Five patients did not have enough evidence for probable or proven invasive fungal disease (IFD) after review of the Data Review Committees.

### Population PK (PPK) model.

**FIG 2:**
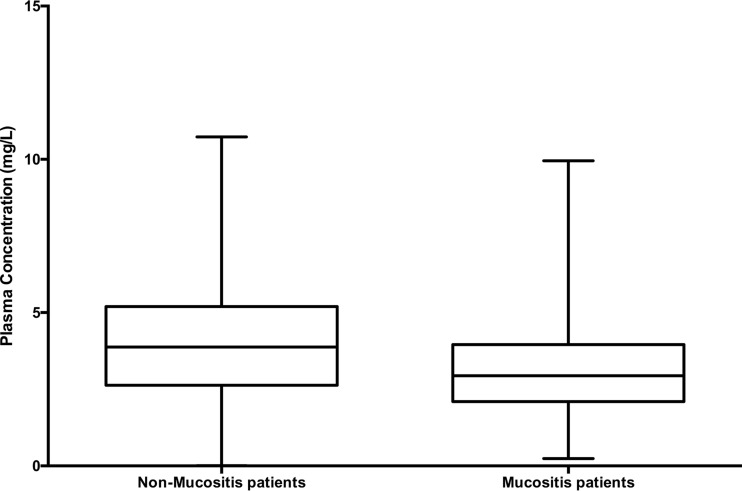
Comparison of plasma concentrations drawn during oral administration after day 7 of therapy between the mucositis and nonmucositis patients. (*P* = 0.0011; Mann-Whitney U test).

**FIG 3:**
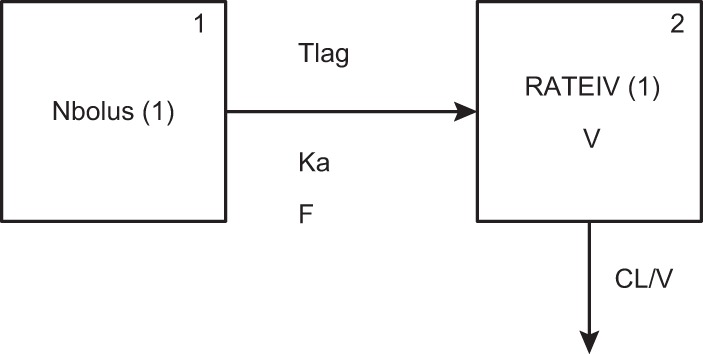
Illustration of the structural model. Compartment 1 represents the gut for oral administration; compartment 2 represents the central compartment. CL, clearance; *F*, bioavailability; Ka, first-order absorption rate constant; Tlag, lag time; *V*, volume in the central compartment. RATEIV (1) specifies infusions going directly into the central compartment.

**FIG 4:**
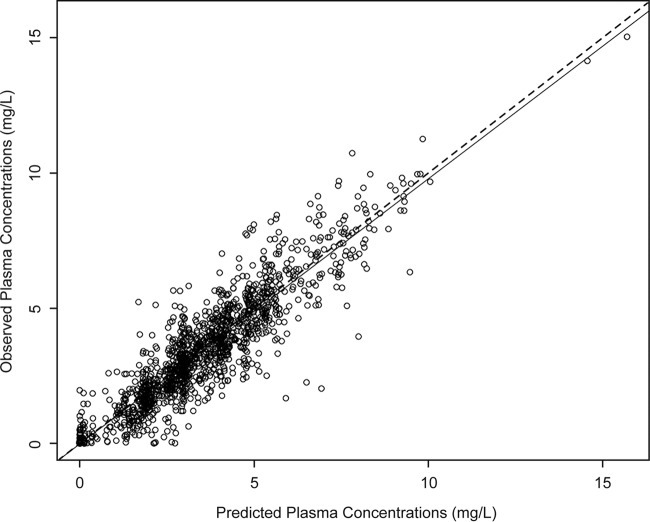
Observed versus median posterior predicted concentrations from the final model after the Bayesian step (*r*^2^ = 0.813; slope = 0.98 [95% CI, 0.956 to 1]; intercept = −0.0181 [95% CI, −0.115 to 0.0792]). The dashed line is a line of unity where observed concentrations equal predicted concentrations.

Comparisons of the raw plasma concentrations for the patients with mucositis and patients without mucositis during oral administration beyond day 7 revealed a statistical difference between the 2 groups (Fig. 2), although the concentrations largely overlapped. A 2-compartment PK model including an absorptive compartment fit the data well. An illustration of the structural model is provided in Fig. 3, where the first compartment represents the gut (oral compartment) and the second represents the central compartment. The fit of the model to the data was acceptable based on visual inspection of the observed-versus-median predicted plots and the coefficient of determination (*r*^*2*^) of 0.813 after the Bayesian step (Fig. 4). The estimates of bias and imprecision were also acceptable (0.11 and 0.938, respectively). The observed-versus-mean predicted plots showed similar statistics, with a coefficient of determination of 0.792 (slope = 0.976) after the Bayesian step. The median parameter estimates are included in Table 2.

**TABLE 2:** Median parameter estimates from the PPK model^*a*^

Parameter	Value for:							
	Mucositis patients				Nonmucositis patients			
	Mean ± SD	Median	Range	% CV	Mean ± SD	Median	Range	% CV
Ka (h^−1^)	7.0 ± 2.6	7.9	0.0–8.0	38	6.5 ± 3.0	7.9	0.0–8.0	46
CL/*F* (liters/h)	2.2 ± 1.0	1.9	0.5–4.1	44	2.3 ± 1.1	1.9	0.1–5.9	47
*V*/*F* (liters)	331.4 ± 154.9	347.7	6.8–895.5	47	354.1 ± 182.5	349.8	5.8–895.5	52
Lag time (h)	1.2 ± 1.2	1.0	0.0–5.0	94	1.3 ± 1.3	1.0	0.0–5.0	103
*F* (%)	86.0 ± 18.5	98.3	50.3–99.7	21	97.4 ± 6.9	99.8	70.2–99.9	7
AUC_ave_ (mg · h/liter)	105.3 ± 55.9	91.9	45.9–315.5	53	114.1 ± 141.2	100.2	30.8–1,944.3	124

a Abbreviations: Ka, first-order absorption rate constant; CL, clearance; *F*, bioavailability; *V*, volume in the central compartment; AUC_ave_, average area under the curve; CV, coefficient of variation.

### Comparison of *F* estimates.

**FIG 5:**
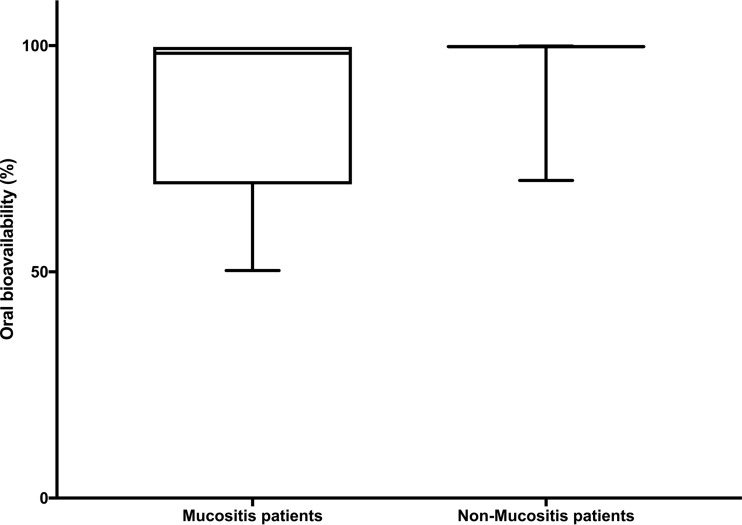
Bioavailability in mucositis and nonmucositis patients. There is a significant difference in the median estimates for bioavailability between the 2 groups. (*P* < 0.0001; Mann-Whitney U test).

**FIG 6:**
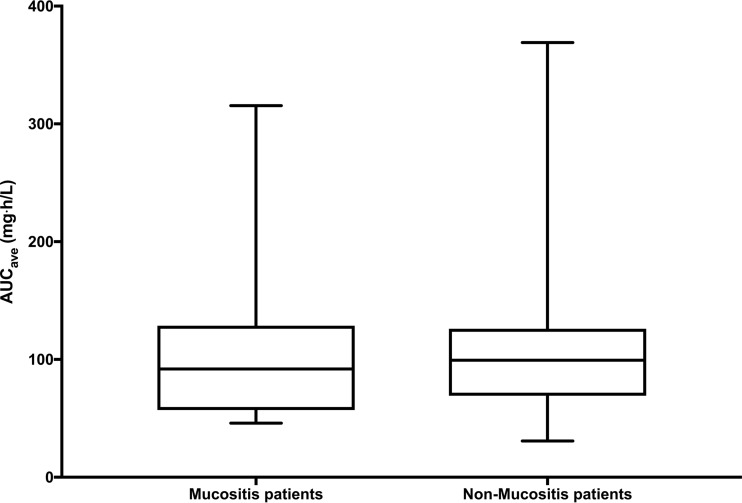
Average AUCs between mucositis and nonmucositis patients. There is no significant difference in average AUCs between the two groups (*P* = 0.706; Mann Whitney U test) (AUCs were calculated from the median parameter estimates after the Bayesian step).

The mean (range) oral bioavailability (*F*) estimates for mucositis and nonmucositis patients were 86.0% (50.3 to 99.7%) and 97.4% (70.2 to 99.9%), respectively. Comparison of the mean and median bioavailability estimates for the two populations demonstrated a significant difference between the 2 groups (*P* < 0.001) (Fig. 5). However, this 11.4% difference in bioavailability did not have a significant impact on the distribution of drug exposures (average area under the curve [AUC_ave_]) between the two groups (*P* = 0.706) (Fig. 6).

### All-cause mortality through day 42.

All-cause mortality rates through treatment day 42 for the patients with and without mucositis were 7.1% (4/56) and 14.4% (28/194), respectively. The oral bioavailability and AUC_ave_ were 83.6% and 91.3 mg · h/liter, 92.7% and 164.9 mg · h/liter, 99.7% and 56.5 mg · h/liter, and 99.7% and 216.9 mg · h/liter for the four patients with mucositis who died. The median bioavailability estimates for the nonmucositis patients that died were all above 90% except for one patient with an estimate of 70.2%. The mean AUC_ave_ was 100.5 mg · h/liter and ranged from 34.9 to 369.1 mg · h/liter.

### Overall response at the EOT.

Overall response at the end of therapy (EOT) was available for 232 mITT (modified intent to treat population) patients in the analysis. Fifty-eight percent (*n* = 43; 95% confidence interval [CI], 42.13, 72.99) and 42.9% (*n* = 189; 95% CI, 35.68, 50.42) of the patients with and without mucositis had a successful response, respectively. In the mucositis patients who failed at the EOT (*n* = 25), the mean oral bioavailability was 84.9% ± 17.9% (range, 50.4 to 99.7%; median, 90.3%) and the mean AUC_ave_ was 117.9 ± 69.4 mg · h/liter (range, 45.9 to 315.5 mg · h/liter; median, 94.2 mg · h/liter). Six of the patients (*n* = 18; 33%) who failed at the EOT had oral bioavailability estimates of <80% (range, 50.4 to 69.5%), with AUC_ave_ values ranging from 45.9 to 176.3 mg · h/liter, and 8 of the patients (*n* = 25; 32%) with successful responses at the end of therapy had bioavailability estimates of <80% (range, 50.3 to 75.5%), with AUC_ave_ ranging from 48.2 to 155.2 mg · h/liter.

## DISCUSSION

Biological factors that have an impact on drug absorption include the pH along the GI tract, tissue perfusion, the presence of bile and mucus, the surface area per volume of the lumen, and the epithelial integrity. Mucositis manifests as erythema, inflammation, ulcerations, and hemorrhage of the mucosal surfaces of the GI tract and causes gastric motility dysfunction. This mucosal disruption can significantly affect drug absorption after the oral administration of medications. Using oral medications in the setting of mucositis requires an understanding of the determinants of drug absorption.

Table 3 summarizes the determinants of oral bioavailability for triazole antifungal agents. Isavuconazole and fluconazole have similar characteristics, including the absence of clinically relevant effect on absorption from food, changes in pH, or increases in GI motility (3, 11, 12). Posaconazole and itraconazole oral solutions should be administered with high-fat meals, carbonated soda, or nutritional supplements (2, 13–18). Plasma concentrations are decreased when gastric acidity is reduced (2, 13–18). Absorption of posaconazole oral solution may be improved when daily doses are more fractionated (19). The newer posaconazole tablets are not affected by changes in gastric pH, and absorption is not improved by the consumption of high-fat meals (16, 20). Plasma voriconazole concentrations are reduced when this drug is taken with food; however, absorption is not clinically significantly affected by changes in pH or by drugs such as omeprazole (21). H_2_ blockers were not found to cause clinically significant changes in voriconazole absorption kinetics (22). Voriconazole exhibits decreased oral bioavailability in patients with cystic fibrosis (CF) compared to that in patients without CF after lung transplantation (23). Thus, factors that affect the absorption of triazoles, such as mucositis, differ markedly.

**TABLE 3:** Comparison of factors impacting oral absorption of triazole antifungal drugs^*a*^

Factor	Datum for drug						
	Isavuconazonium sulfate	Voriconazole	Posaconazole (14)		Itraconazole (15)		Fluconazole (12)
Formulation	Capsule	Tablets	Solution	Tablets	Solution	Capsule	Tablet
Water solubility	Y (prodrug)	N	N	N	N	N	Y
Bioavailability (%)							
Healthy subjects	98 (11)	96 (29)	8–48 (fasted)	54 (fasted)	55		90
Patients	97 (5)	64 (30)					
GI motility agents	None	No data found	Decreases	None	No data found		No data found
pH effect	None	None	Decreases in reduced acidity	None	Decreased in reduced acidity		None
Food effect	None	Decreases concentrations	Increases concentrations (especially high fat, nutritional supplement, or acidic carbonated beverage)	*C*_max_ and AUC increases 16% and 51% with high-fat foods	Increases concentrations		None
Other		*F* significantly lower in CF lung tx (23%) pts vs non-CF lung tx (63%) (23); 2 factors with significant association with *F* in lung tx pts: CF, postoperative time (increased with increasing time) (23)	Divided doses increase absorption				
Substrate of Pgp	N	N	Y	Y	Y	Y	N

a Abbreviations: Y, yes; N, no; GI, gastrointestinal; *F*, bioavailability; CF, cystic fibrosis; Pgp, P-glycoprotein; *C*_max_, maximum concentration; tx pts, transplant patients.

Drugs that require food to increase oral bioavailability or experience decreased bioavailability with increased gastric emptying (increased gastric motility) suggest that passive diffusion is slow and likely occurs primarily from the stomach. In these circumstances, absorption is improved by longer transit times in the stomach and upper small intestine. Aside from the prodrug formulation of isavuconazole, the other azoles are limited by the insufficient dissolution in stomach prior to delivery in the duodenum, where absorption is maximal. A meal that is high in fat increases luminal volume and bile and pancreatic secretions and delays gastric emptying. The absorption for drugs such as posaconazole may be optimized by the use of a more fractionated regimen (13, 24, 25). However, studies have suggested that this may be due to the high-fat meal increasing the solubility versus delayed gastric emptying (13). Another study failed to associate factors such as P-glycoprotein with the absorption of posaconazole (26). In contrast, the absorption for isavuconazole and fluconazole (and, to a lesser extent, voriconazole) is not significantly influenced by these factors, suggesting that passive diffusion occurs more quickly and the majority of the absorption occurs in the upper small intestine.

In this analysis, the presence of mucositis did not have a significant overall impact on the clinical outcomes in the patients treated with isavuconazonium sulfate from the SECURE and VITAL trials despite the statistical differences in oral bioavailability between the groups with and without mucositis. In addition, the drug exposures between the groups were not significantly different. The results were similar regardless of whether mean or median parameter estimates were used for the comparisons.

The current study has several limitations. First, details on the presence or severity of mucositis were not available for the majority of patients with the condition. Quantification of severity may have allowed for a deeper understanding of the impact for the degree of mucosal disruption and the impact on oral bioavailability. Second, patients were allowed to switch back and forth from oral to intravenous (i.v.) medication during the treatment period. However, only patients with mucositis coinciding with oral administration were selected for analysis. Third, the administration of i.v. or oral formulations was at the discretion of the site investigators, making it difficult to assess the impact of the severity of mucositis on oral bioavailability. Patients with more severe grades of mucositis patients may have remained on i.v. therapy longer, while patients with less severe mucositis may have been switched to oral therapy. In addition, identification of mucositis patients for this study relied on the reporting of the events by the treating investigator, which could be underrepresenting the incidence in the study. We did not utilize a validated mucositis score or a biomarker, such as citrulline, to capture severity as has been done in other studies (27). Finally, we assumed that compliance was 100%, which may be overly optimistic.

These analyses are important, as many patients who will be treated with isavuconazonium sulfate are at risk or could have mucositis at the onset of therapy caused by the harsh treatments used for their underlying comorbidities. Patients with slightly lower bioavailability had outcomes similar to those of patients with higher bioavailability. Therefore, use of the oral formulation of isavuconazonium sulfate during episodes of mucositis may be acceptable; however, treating physicians may consider extending isavuconazole intravenous therapy during episodes of mucositis or monitoring levels to ensure that they are within the range reported from the clinical trial. However, additional studies with this population may be warranted.

## MATERIALS AND METHODS

### Study design.

Patients treated with isavuconazonium sulfate from two phase 3 clinical trials, SECURE and VITAL, were eligible for inclusion if plasma concentrations were available. The SECURE trial (ClinicalTrials.gov identifier: NCT00412893) evaluated the efficacy and safety of isavuconazole compared with voriconazole for the primary treatment of invasive mold disease caused by Aspergillus spp. and other filamentous fungi (9). The VITAL trial (ClinicalTrials.gov identifier: NCT00634049) evaluated the efficacy and safety of isavuconazole for the treatment of IA in patients with renal impairment and in patients with IFD caused by Mucorales and other emerging molds, yeasts, and dimorphic fungi (10). Eligibility criteria for both studies are detailed elsewhere (9, 10). Patients received a loading regimen of isavuconazonium sulfate at a dose of 372 mg (equivalent to isavuconazole 200 mg) every 8 h for the first 48 h. In the SECURE trial, the loading dose was required to be administered intravenously (i.v.), while in the VITAL trial, treatment could commence using either the i.v. or oral formulation. The maintenance regimen for both studies was i.v. or oral isavuconazonium sulfate at 372 mg once daily for up to 84 or 180 days, respectively. Patients received i.v. or oral drug at the discretion of site investigators.

### Identification of patients with mucositis.

The medical history (MH) and adverse event (AE) records from the case report forms were reviewed for *Medical Dictionary for Regulatory Activities*-preferred terms suggestive of “mucositis” or “stomatitis” (e.g., mucosal inflammation, radiation mucositis, stomatitis, and gastrointestinal inflammation). From there, the patients were further reviewed to determine the degree of likelihood that the MHs and AEs reported represented significant disease, such as recent radiation therapy or intensive chemotherapy. Patients with mucositis were included only if administration of the oral formulation occurred during the episode of mucositis and plasma PK concentrations coincided with the oral administration and episode of mucositis. Patients without mucositis with plasma PK measurements during oral administration were classified as nonmucositis patients.

### Plasma PK sampling.

Blood samples were collected on treatment days 7, 14, and 42 and at the end of therapy (EOT) in both trials. Collection was targeted for 24 h after the start of the infusion or the oral dose on the previous day (i.e., trough concentration). Full 24-h profiles were obtained from a subset of 43 patients (including 6 patients with mucositis). After collection, samples were processed immediately and stored at −80°C until shipment to the central research laboratory. Isavuconazole concentrations were measured at the completion of the study using a validated liquid chromatography-tandem mass spectrometry (LC-MS/MS) method as previously described (5).

### PPK modeling.

Raw plasma concentration data from the 2 groups during oral administration that were collected after day 7 were compared to determine if any trends in the data could be observed. A population PK (PPK) model was developed using nonparametric estimation using Pmetrics (v1.4.1; University of Southern California, Los Angeles, CA) (28). The model-fitting process included evaluation of both 2- and 3-compartment models including absorptive compartments and a lag time. The presence of mucositis (yes = 1; no = 0) was used as a covariate on oral bioavailability (*F*) as a secondary equation, which took the following form: *F* = *F*1 × (1 − MUC) + *F*12 × MUC, where *F*1 refers to the oral bioavailability in patients without mucositis (MUC = 0) and *F*12 refers to the oral bioavailability in patients with mucositis (MUC = 1).

Data were weighted by the inverse of the estimated assay variance. The final model was assessed by a visual inspection of the observed-versus-predicted concentration values before and after the Bayesian step, the coefficient of determination (*r*^*2*^) from the linear regression of the observed-versus-predicted values, as well as estimates for bias (mean weighted error) and precision (adjusted mean weighted squared error).

The average AUC (AUC_ave_) for each patient was calculated using the Bayesian posterior parameter estimates from the final model using the trapezoidal rule in Pmetrics. AUC_ave_ was calculated by determining the total AUC over the entire dosing period and dividing by the number of days of therapy for each patient. Statistical comparisons were performed in MYSTAT 12 version 12.02 (Systat) and GraphPad Prism version 6.0h (GraphPad).

### Exposure-response analysis.

The AUC_ave_ values for patients with and without mucositis were compared by patient outcomes defined as all-cause mortality through day 42 or overall response to explore if any impact on exposure was associated with differences in response. Statistical comparisons were performed in MYSTAT 12 (version 12.02; Systat).
